# Experimental models in diabetes research

**DOI:** 10.1186/s42826-026-00286-6

**Published:** 2026-07-02

**Authors:** Lalit P. Dewalkar

**Affiliations:** Department of Zoology, Guru Nanak College of Science, Affiliated with Gondwana University Gadchiroli, Ballarpur, Maharashtra 442701 India

**Keywords:** Diabetes mellitus, Disease models, Insulin resistance, Pancreatic β-cells, Translational medical research

## Abstract

Diabetes mellitus is a major global health challenge, affecting 11.1% of adults worldwide, with nearly half of the cases remaining undiagnosed. Despite extensive progress, its multifactorial pathogenesis requires further investigation to advance therapeutic development. Experimental models are indispensable for understanding disease mechanisms and evaluating interventions, although no single model fully recapitulates human diabetes. Chemical agents such as streptozotocin and alloxan simulate β-cell loss, whereas dietary and obesity-induced models reflect insulin resistance and metabolic disturbances. Genetic models, including *ob/ob*, *db/db*, and non-obese diabetic mice, provide insights into obesity-associated and autoimmune pathways, whereas in vitro systems enable controlled mechanistic studies and drug screening. This review integrates evidence from diverse experimental platforms, highlighting their comparative strengths, limitations, and translational applicability to support rational model selection and enhance the efficiency of diabetes research and therapeutic innovation.

## Background

Diabetes has become a widespread health challenge, affecting the lives of countless individuals across the globe. According to the latest 11th Edition (2025) of the International Diabetes Federation (IDF) Diabetes Atlas, the global age-standardized prevalence of diabetes among adults aged 20–79 years is estimated to be 11.1%, corresponding to approximately 1 in 9 adults living with the condition. Alarmingly, more than 43% of these individuals remain undiagnosed, highlighting the substantial hidden burden of this disease worldwide [1]. The IDF reported that global diabetes related health expenditure will reach approximately USD 1 trillion in 2024, reflecting a 338% increase over the past 17 years [1]. This situation is expected to worsen, particularly in underdeveloped and developing countries. In these regions, nearly half of individuals with diabetes are unaware of their condition, making prevention and treatment challenging [1, 2]. This lack of awareness and access to healthcare in low- and middle-income countries highlights the urgent need for improved diabetes education, screening, and care on a global scale.

Type 1 diabetes mellitus (T1DM) and type 2 diabetes mellitus (T2DM) are represent the most common forms of diabetes. T1DM occurs when the immune system mistakenly attacks and destroys the insulin producing β-cells in the pancreas [3]. This leaves the body unable to produce its own insulin, a crucial hormone for controlling blood sugar levels [4]. T2DM, on the other hand, develops gradually. Insulin resistance is characterized by a reduced responsiveness of the body’s cells to insulin, thereby complicating the regulation of blood glucose levels. Concurrently, the pancreas encounters difficulty in producing sufficient insulin to counteract this resistance. This form of diabetes is frequently associated with lifestyle choices and genetic predispositions [3, 5]. Several factors can elevate the risk of developing T2DM, including being overweight, insufficient physical activity, and a diet high in calories. Additionally, certain individuals may have a heightened likelihood of developing T2DM due to their genetic composition [6, 7].

The development of T2DM is attributed to a complex interplay of cellular-level factors. These factors encompass impairments in insulin signaling, lipid-induced damage, stress induced by reactive oxygen species, endoplasmic reticulum (ER) stress, mitochondrial dysfunction, chronic low-grade inflammation, and altered adipokine signaling [5–8]. In contrast, T1DM is predominantly influenced by an individual’s genetic composition, particularly specific immune system genes. Additionally, environmental factors such as viral infections, dietary components, and gut microbiota can precipitate an autoimmune response, leading to the erroneous destruction of insulin-producing cells in the pancreas [9–12].

Given the complexity and multifactorial nature of diabetes, experimental models remain indispensable tools for unraveling its underlying mechanisms and for testing potential therapies. Although no single system can capture the full spectrum of human diabetes, a diverse array of in vivo and in vitro models allows us to dissect critical aspects such as β-cell dysfunction, insulin resistance, metabolic disturbances, molecular signaling, and compensatory responses. This review discusses the major experimental approaches, chemical, dietary, genetic, surgical, and cell-based models highlighting their respective strengths, limitations, and contexts of use. By critically evaluating these models, this review aims to guide researchers in choosing the most appropriate systems for their studies, ultimately enhancing experimental design and sharpening the translational impact of diabetes research.

## Main text

### Literature search strategy

A narrative literature search was conducted using databases such as PubMed, Scopus, and Web of Science to identify peer-reviewed articles discussing experimental models used in diabetes research, including both in vitro and in vivo systems; keywords such as diabetes mellitus, type 1 diabetes, type 2 diabetes, experimental model, animal model, cell line, and insulin resistance were used with Boolean combinations, and reference lists of key studies were screened for additional sources; eligible articles were those describing the development, characterization, or application of diabetes models, while non-scientific reports, abstracts without full text, and exclusively clinical studies were excluded; relevant data were extracted and thematically organized to summarize model types, induction techniques, physiological relevance, advantages, and limitations for effective comparison and research applicability.

### In vivo models of diabetes

In vivo models effectively replicate the intricate characteristics of diabetes observed in humans, providing significant insights into disease progression and potential therapeutic interventions. These models enable researchers to observe and manipulate conditions analogous to diabetes, thereby elucidating the underlying mechanisms and evaluating novel therapeutic strategies within a controlled environment. Based on a literature survey, these models can be broadly classified into diet-induced, chemically induced, genetic, and surgical models. Chemically induced and surgical models are particularly useful for studying insulin deficiency (T1DM), whereas diet-induced and genetic models more closely mimic obesity-associated insulin resistance and metabolic dysregulation (T2DM). The selection of an appropriate model depends on the specific research question, which can range from β-cell biology to metabolic syndrome and immunological mechanisms.

#### Diet induced models

Diet-induced models are employed to replicate the metabolic disturbances observed in humans, including obesity, insulin resistance, dyslipidemia, low-grade inflammation, and hyperglycemia [13]. These models are particularly valuable for investigating the pathogenesis of T2DM and its associated complications in humans. The high-fat diet (HFD) model for inducing a T2DM like state in laboratory mice was first systematically described by Surwit et al. in the late 1980s, who demonstrated that feeding C57BL/6J mice a diet high in fat and sucrose led to the development of obesity, hyperinsulinemia, insulin resistance, and hyperglycemia, closely resembling human T2DM pathogenesis [14–16].

**Fig. 1:**
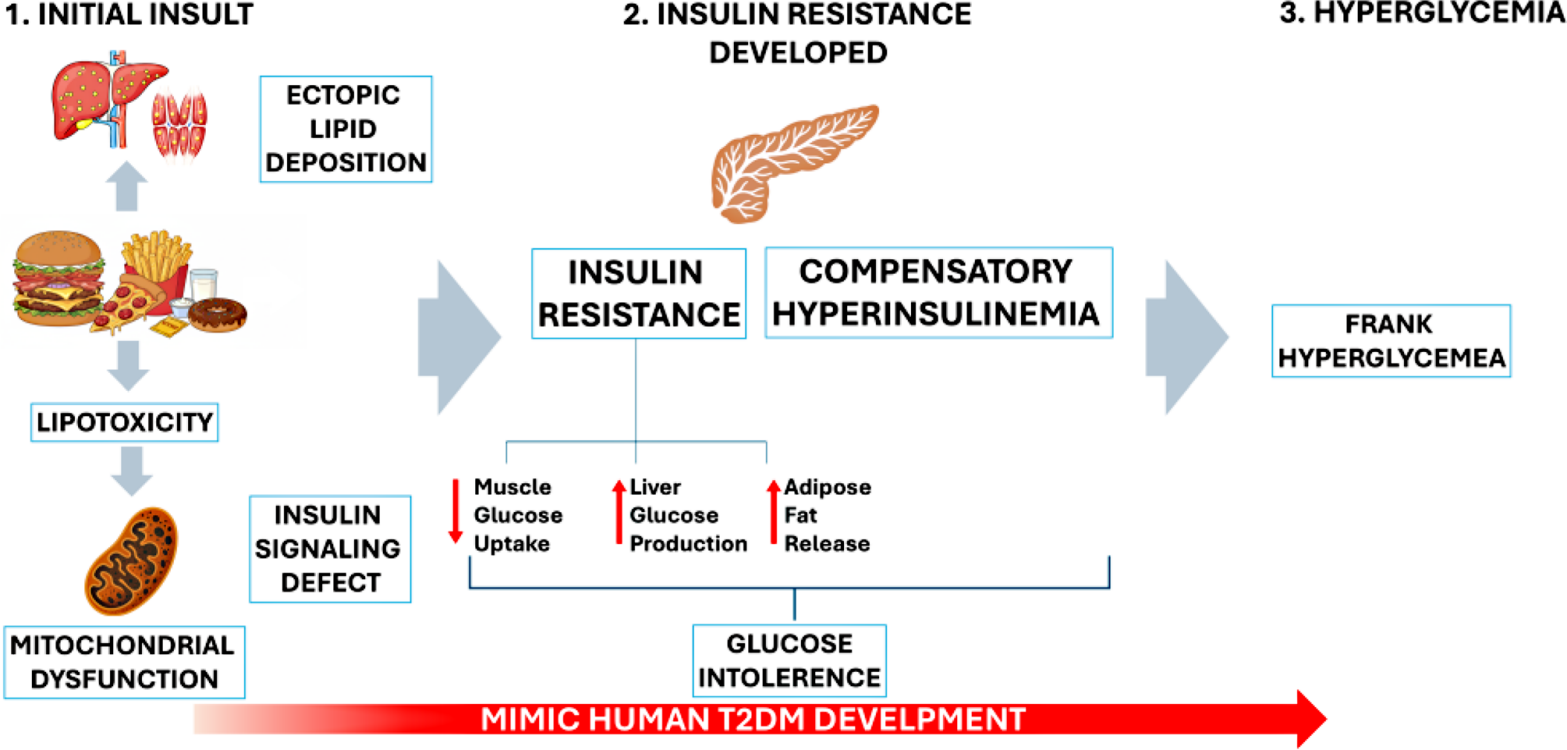
Hight fat diet intake induces ectopic lipid deposition, lipotoxicity, and mitochondrial dysfunction, leading to insulin resistance and compensatory hyperinsulinemia. Progressive insulin resistance results in glucose intolerance and ultimately frank hyperglycemia, mimicking the development of human T2DM [14, 17, 18]

Prolonged intake of a diet high in fat content (typically comprising 45–60% of caloric intake from fat) results in an energy surplus, which subsequently leads to the expansion of adipose tissue, ectopic lipid accumulation (such as in the liver and muscle), mitochondrial dysfunction, lipotoxicity and impaired insulin signaling (Fig. 1) [17, 19]. Over time, the metabolic burden leads to insulin resistance in peripheral tissues (muscle, adipose, and liver), compensatory hyperinsulinemia, and eventually glucose intolerance and frank hyperglycemia in susceptible strains [18, 20]. This progression mimics the development of human T2DM in many respects (Fig. 1). Parallel developments included high-sucrose and high-fructose diet models, which provided insights into hepatic insulin resistance, dyslipidemia, and non-alcoholic fatty liver disease (NAFLD), even in the absence of obesity [21–23].

Over time, combination models such as high-fat, high-sucrose diets (HFHS) or high-fat, high-fructose diets (HFFD), in addition to chemicals such as streptozotocin (STZ), have been introduced to accelerate disease onset and replicate the multifactorial nature of human metabolic diseases [24–26]. Diet-induced models are integral to preclinical diabetes research. Surwit’s seminal HFD paradigm established the groundwork for this experimental methodology, facilitating a deeper understanding of the intricate relationship between diet and diabetes mellitus. This approach has enabled researchers to replicate human dietary patterns and their impact on metabolic health in laboratory environments, advancing our understanding of the mechanisms underlying diet-related diabetes and informing the development of potential interventions. A comparative summary of the major diet-induced and combination models, including their mechanisms, metabolic characteristics, advantages, and limitations, is provided in Table 1.

**Table 1:** Comparative overview of dietary and combination experimental models of type 2 diabetes mellitus. The table summarizes commonly used rodent models, their underlying mechanisms, characteristic metabolic features, advantages, and limitations

Model	Mechanism	Key Features	Advantages	Limitations	References
High Fat Diet	Excess fat intake causes adiposity, lipotoxicity, insulin signaling defects	Obesity, insulin resistance, hyperinsulinemia, impaired glucose tolerance	Mimics gradual human T2DM progression; widely used; tunable severity	Time-consuming (8–20 weeks); strain/sex variability; not always overt diabetes.	[14–16]
High Sucrose / Fructose Diet	Excess sugar leads to hepatic de novo lipogenesis, oxidative stress, insulin resistance	Hepatic insulin resistance, dyslipidemia, metabolic syndrome, sometimes hypertension	Rapid onset; useful for prediabetes and liver metabolism studies	Often lacks obesity; effects depend on species and sex; non-physiological sugar doses.	[21–23]
Combination Models (HFHS+HFFD + STZ)	Synergistic metabolic and β-cell stress	Insulin resistance and β-cell dysfunction induces T2DM	Produces robust diabetic phenotype; closer to human T2DM	Complex protocols; higher morbidity; requires careful titration.	[24–26]

### Chemically induced models

#### Alloxan induced diabetic model

**Fig. 2:**
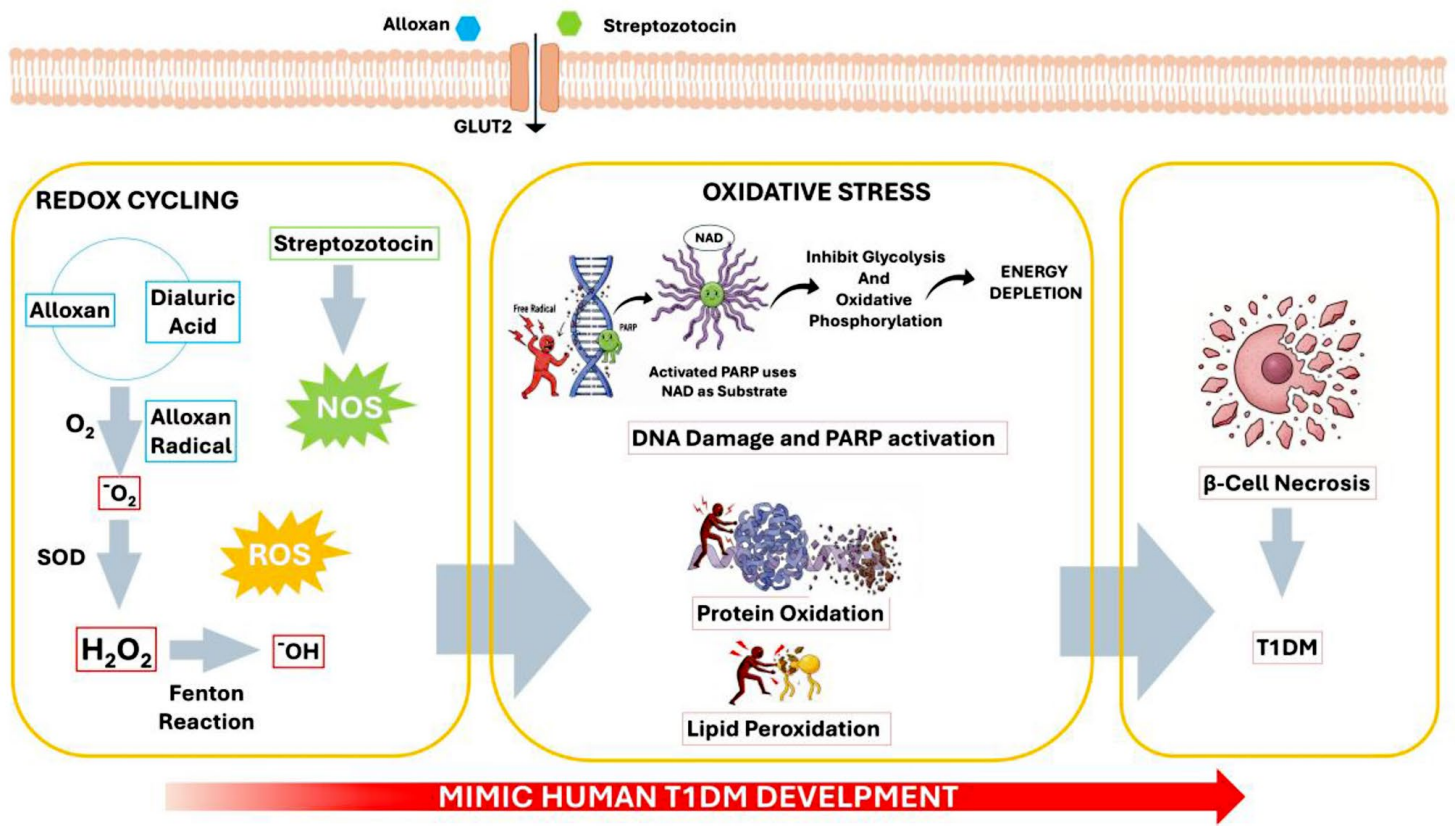
Mechanism of alloxan and streptozotocin-induced type 1 diabetes. Both agents selectively enter pancreatic β-cells via GLUT2 and undergo redox cycling, leading to excessive ROS generation. Resulting oxidative stress triggers lipid, protein, and DNA damage, PARP activation, and depletion of NAD⁺ and ATP, ultimately causing irreversible β-cell destruction, insulin deficiency, and sustained hyperglycemia [38–41]

Alloxan is a widely used diabetogenic chemical agent for inducing experimental T1DM in laboratory animals because of its selective cytotoxicity toward pancreatic β-cells [27–30]. Following administration, alloxan is selectively taken up by β-cells via the GLUT2 glucose transporter, which is abundantly expressed in rodents [31, 32]. Once inside the cell, alloxan undergoes redox cycling, resulting in the formation of dialuric acid, which leads to the generation of reactive oxygen species (ROS), such as superoxide anion (O₂⁻), hydrogen peroxide (H₂O₂), and highly reactive hydroxyl radicals (^−^OH), which induce oxidative stress and subsequent damage to cellular macromolecules [33–35]. This process results in the activation of PARP, depletion of NAD⁺/ATP, disruption of calcium homeostasis, and ultimately β-cell necrosis, which leads to insulin deficiency and hyperglycemia (Fig. 2) [36–38]. The culmination of these events leads to irreversible β-cell destruction, insulin deficiency, and persistent hyperglycemia, effectively mimicking the pathophysiology of human T1DM [28, 33, 39, 40].

#### Streptozotocin-induced diabetic model

Streptozotocin (STZ), a glucosamine-conjugated nitrosourea compound, is another widely used β-cell–specific cytotoxic agent that enters β-cells primarily via GLUT2 [32, 42, 43]. Unlike alloxan, STZ acts mainly as a DNA-alkylating agent, triggering severe DNA strand damage, PARP-driven NAD⁺ and ATP exhaustion, and a β-cell energy crisis [44–46]. Additionally, its nitrosourea group liberates nitric oxide (NO), impairing mitochondrial oxidative metabolism and enhancing β-cell death [47–49]. Depending on the dose and regimen, STZ can be used to induce T1DM through a single high-dose injection or a more gradual autoimmune-like β-cell destruction resembling T1DM via multiple low-dose (MLD-STZ) protocols [50]. Furthermore, when combined with HFD, STZ is employed to establish T2DM models that better reflect the dual pathology of insulin resistance and β-cell dysfunction [28, 39].

Both alloxan and STZ rely on GLUT2-mediated uptake for β-cell selectivity; however, GLUT2 expression is markedly higher in rodent β-cells than in human cells, where GLUT1 predominates [51]. This interspecies difference can alter cytotoxic sensitivity and limit the direct translational applicability to human β-cell physiology and therapeutic prediction. Therefore, although chemically induced models are valuable for mechanistic and pharmacological studies, extrapolation to human diseases must be performed with caution.

#### MSG-induced diabetic model

**Fig. 3:**
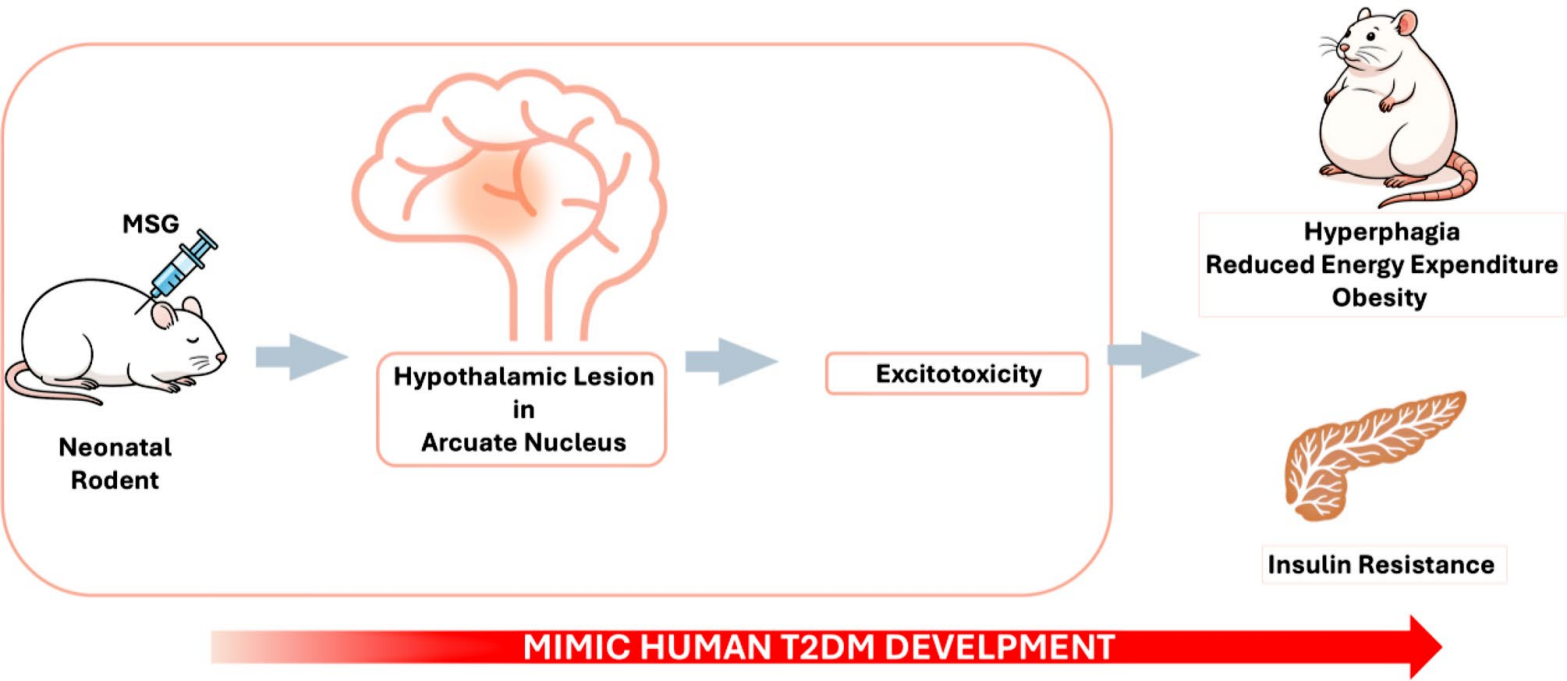
Neonatal exposure to monosodium glutamate (MSG) causes hypothalamic lesions via excitotoxicity, leading to impaired leptin and insulin signalling, hyperphagia, obesity, insulin resistance, and metabolic disturbances that culminate in a T2DM-like phenotype

Monosodium glutamate (MSG) administration in neonatal rodents has been used as a model to study obesity, insulin resistance, and T2DM like metabolic disturbances (Fig. 3). Neonatal exposure to MSG causes lesions in the hypothalamic arcuate nucleus, particularly in the regions regulating satiety and energy homeostasis, such as the arcuate nucleus (ARC) and ventromedial hypothalamus (VMH) [52, 53]. This neurotoxic effect is primarily mediated through excitotoxicity due to excessive glutamate signaling, leading to impaired leptin and insulin signaling pathways in the central nervous system [54, 55].

As a consequence of hypothalamic damage, animals exhibit hyperphagia, reduced energy expenditure, and obesity, followed by insulin resistance, hyperinsulinemia, dyslipidemia, and impaired glucose tolerance [56, 57]. Over time, this metabolic imbalance progresses toward a T2DM-like phenotype. Importantly, MSG-induced obesity is characterized by an increased deposition of visceral adipose tissue and associated low-grade inflammation, which contribute significantly to insulin resistance (Fig. 3) [57, 58].

The MSG model has several advantages, including noninvasive induction, ability to replicate obesity-associated insulin resistance, and suitability for studying metabolic syndrome and T2DM pathophysiology [59]. However, limitations include species- and strain-specific sensitivity to MSG, neurotoxic side effects, and variability in the extent of diabetes development depending on dose and age at administration [57, 60, 61].

### Genetic model for diabetes

Genetic models of diabetes shed light on how inherited factors drive the disease. By mirroring the diverse predispositions seen in people, researchers can follow the pathways leading to both type 1 and type 2 diabetes. Just as no two individuals experience diabetes in the same way, these models reflect the genetic diversity behind the condition and support the search for more personalized and effective treatments. These models encompass both spontaneous mutations and inbred strains carrying defined genetic defects, offering insights into how inherited factors contribute to diabetes (Table 2).

**Table 2:** Key characteristics of widely used genetic rodent models of diabetes. These models differ in genetic background, obesity status, and metabolic features, thereby mimicking distinct aspects of human diabetes

Model	Genetic Defect	Obesity Status	Key Metabolic Features	Major Complications	Research Applications	References
NOD Mouse	Polygenic, autoimmune susceptibility loci (e.g., MHC class II I-Ag7)	Non-obese	Autoimmune destruction of pancreatic β-cells, insulin deficiency, spontaneous T1DM	Nephropathy, neuropathy, retinopathy, insulitis	Type 1 diabetes pathogenesis, autoimmune studies, immunotherapy development	[62–65]
*ob/ob* Mouse	Mutation in leptin gene → leptin deficiency	Obese	Hyperphagia, obesity, insulin resistance, hyperinsulinemia, mild to moderate hyperglycemia	Limited complications unless aged or stressed	Obesity research, leptin biology, early T2DM	[66–69]
*db/db* Mouse	Mutation in leptin receptor gene (autosomal recessive)	Obese	Hyperphagia, hyperinsulinemia, insulin resistance, progressive hyperglycemia, dyslipidemia	Nephropathy, retinopathy, neuropathy, cardiomyopathy	Obesity-associated T2DM, diabetic complications, antidiabetic drug testing	[67, 70–73]
Zucker Fatty Rat (*fa/fa*)	Missense mutation in leptin receptor gene	Obese	Hyperphagia, hyperlipidemia, insulin resistance, mild hyperglycemia, compensatory hyperinsulinemia	Fatty liver, renal injury, hypertension, metabolic syndrome features	Obesity, metabolic syndrome, drug development (anti-obesity, insulin sensitizers)	[74–78]
Goto-Kakizaki (GK) Rat	Polygenic, from selective inbreeding of Wistar rats with impaired glucose tolerance	Non-obese	Impaired glucose tolerance, β-cell dysfunction, insulin resistance, reduced β-cell mass	Nephropathy, neuropathy, retinopathy, cardiovascular complications	Non-obese T2DM, β-cell dysfunction, long-term drug studies	[79–82]

#### Non-obese diabetic mouse

The Non-Obese Diabetic (NOD) mouse is one of the most widely used and well-characterized genetic models for T1DM (Table 2). This strain develops spontaneous autoimmune diabetes, with a pathogenesis that closely mirrors the human disease. In NOD mice, autoreactive CD4⁺ and CD8⁺ T- lymphocytes infiltrate the pancreatic islets (insulitis), leading to progressive β-cell destruction, insulin deficiency, and hyperglycemia [62–64]. Disease onset typically occurs between 12 and 30 weeks of age, with females showing a higher incidence than males [65]. At the genetic level, NOD mice carry multiple insulin-dependent diabetes (Idd) susceptibility loci, including those that overlap with human HLA gene regions, which are critical for immune tolerance and β-cell autoimmunity [65]. Environmental influences such as microbial exposure, diet, and housing conditions also modulate disease incidence, highlighting the interplay between genetic predisposition and environmental triggers [83]. The NOD model has also been pivotal in uncovering genetic modifiers of diabetes susceptibility. Recently, Hervé et al. (2024) described a novel NOD subline, termed “HYP”, characterized by a spontaneous Akt2 deficiency [84]. This defect accelerates β-cell dysfunction, leading to early hyperglycemia and faster diabetes onset, thus providing new insights into how genetic alterations can modulate disease phenotype and progression.

The NOD mouse has been instrumental in dissecting the immunological mechanisms underlying T1DM, including the roles of autoreactive T-lymphocytes, B-lymphocytes, dendritic cells, and macrophages [29, 30, 62]. The NOD mouse remains valuable resource for diabetes research, providing a preclinical platform to evaluate potential therapies before they are tested in humans. Studies using this model have enabled the development and refinement of innovative approaches, including immunotherapies that target autoreactive lymphocytes, interventions designed to promote immune tolerance to β-cell antigens, and regenerative strategies aimed at replacing lost insulin-producing cells. By first validating these treatments in NOD mice, researchers can more efficiently identify promising candidates while also assessing safety and efficacy, thereby accelerating the translation of experimental therapies into clinical studies [65]. While the autoimmune characteristics are of significant relevance, certain limitations exist. Differences in immune regulation and the relatively rapid progression compared to humans may complicate translational applications [85].

#### ob/ob mouse

The *ob/ob* mouse represents one of the earliest and most extensively investigated genetic models of obesity-associated type 2 diabetes mellitus (T2DM). This model harbors a spontaneous mutation in the leptin gene (Lep), resulting in a complete deficiency of leptin, a hormone integral to the regulation of satiety, energy expenditure, and glucose metabolism [67, 86, 87]. The diabetic phenotype is contingent upon the genetic strain; for instance, *ob/ob* mice on the C57BL/6J background exhibit significant obesity and metabolic dysfunction, whereas those on the C57BLKS/J background manifest more severe and persistent diabetes due to heightened β-cell dysfunction [68, 88–90].

This model has been widely employed to study energy balance, adipose biology, leptin signaling pathways, obesity-induced insulin resistance, and diabetes complications [66, 69, 91]. However, its translational relevance is limited by the rarity of congenital leptin deficiency in humans; most cases of human T2DM arise from polygenic factors and environmental influences rather than leptin deficiency [92, 93].

#### db/db mouse

The *db/db* mouse is a widely used genetic model for T2DM and obesity research. This model carries a spontaneous autosomal recessive mutation in the leptin receptor gene located on chromosome 4, which leads to impaired leptin signaling and an inability to regulate appetite and energy expenditure [70, 71]. The diabetic phenotype typically manifests at 4–8 weeks of age, accompanied by dyslipidemia, hepatic steatosis, and an increased risk of diabetic complications, such as nephropathy, retinopathy, and cardiomyopathy [73, 94].

Owing to their close resemblance to human obesity-associated T2DM, *db/db* mice have been extensively employed in research focused on the pathophysiology, drug development, and complications of diabetes. They are particularly valuable for evaluating the efficacy of antidiabetic agents, including insulin sensitizers, GLP-1 receptor agonists, and DPP-4 inhibitors [95, 96]. However, limitations exist, as severe obesity in this model may obscure diabetes-specific metabolic effects, and differences in rodent and human β-cell physiology restrict direct translational extrapolation [97].

#### Zucker fatty rat (fa/fa)

The Zucker fatty (*fa/fa*) rat is a classic genetic model of obesity and T2DM that was first described in the 1960s [74]. These rats carry a homozygous missense mutation in the leptin receptor gene, which disrupts leptin signaling. As a result of impaired leptin signaling, *fa/fa* rats develop obesity associated with glucose intolerance. However, in contrast to *db/db* mice, the severity of hyperglycemia in *fa/fa* rats is generally milder and more variable, reflecting differences in β-cell compensation and genetic background [76, 77].

The *fa/fa* phenotype becomes apparent as early as 3–5 weeks of age, when Zucker fatty rats display rapid weight gain driven by hyperphagia and reduced energy expenditure [76, 77]. In addition to their metabolic abnormalities, *fa/fa* rats develop dyslipidemia, hepatic steatosis (fatty liver), renal injury, and hypertension, which makes them a valuable experimental model for investigating obesity, metabolic syndrome, and associated complications [98, 99].

Zucker fatty rats are widely used for evaluating antidiabetic and anti-obesity therapies, including insulin sensitizers, leptin analogs, and appetite-regulating agents [100]. However, a limitation of this model is that many *fa/fa* rats maintain compensatory hyperinsulinemia and do not consistently progress to frank diabetes, which distinguishes them from more severe models like the *db/db* mouse [78].

The *ob/ob*, *db/db*, and *fa/fa* rat models exhibit a common mechanistic cascade characterized by disrupted leptin pathway signaling, which leads to hyperphagia, progressive obesity, insulin resistance, and subsequent glucose intolerance [72, 75, 101, 102].

#### Goto-Kakizaki rat

The Goto-Kakizaki (GK) rat is one of the most widely used nonobese genetic models of T2DM. The GK rat was established in Japan in the 1970s by Goto and Kakizaki through the selective inbreeding of Wistar rats that exhibited impaired glucose tolerance [79]. Unlike other genetic models, such as Zucker fatty (*fa/fa*) rats or *db/db* mice, GK rats develop spontaneous T2DM without obesity. This characteristic renders it particularly valuable for investigating the lean T2DM phenotype in humans [80]. GK rats demonstrate defective insulin secretion, insulin resistance, and impaired glucose tolerance from an early age (3–4 weeks). The primary defect is pancreatic β-cell dysfunction, characterized by reduced β-cell mass, impaired insulin biosynthesis, and defective glucose-stimulated insulin secretion [82]. In addition, GK rats develop secondary complications, such as nephropathy, retinopathy, neuropathy, and cardiovascular dysfunction, closely mirroring human diabetic complications [80].

The GK rat is particularly valuable for investigating the pathophysiology of β-cell dysfunction and diabetic complications and evaluating therapeutic interventions in the context of nonobese T2DM. However, important limitations exist: the strain displays genetic heterogeneity and develops only moderate hyperglycemia compared with more severe models, such as Zucker fatty (*fa/fa*) rats or *db/db* mice. These features may restrict its translational applicability in the study of advanced or severe forms of T2DM [80, 81].

### Surgical models

Surgical diabetes models provide valuable insights into the pathophysiology of insulin deficiency and β-cell regeneration. Partial pancreatectomy, which involves the removal of 60–90% of the pancreatic tissue, reduces functional β-cell mass and induces moderate hyperglycemia, thereby mimicking the progressive β-cell loss observed in T2DM [103, 104]. In contrast, total pancreatectomy results in the complete removal of pancreatic β-cells, leading to an absolute insulin deficiency and permanent diabetes. This approach provides a robust and reproducible model of T1DM and is widely used in islet transplantation and β-cell replacement studies [105]. Another surgical approach, pancreatic duct ligation, causes acinar cell atrophy and inflammation, which in turn promotes selective β-cell loss and pancreatic remodeling. This model has been widely employed to investigate the mechanisms of β-cell destruction and regeneration [106]. Although technically demanding and associated with surgical stress, surgical models such as total pancreatectomy and pancreatic duct ligation remain valuable tools for investigating the pathophysiological mechanisms of diabetes that cannot be adequately reproduced by chemical or genetic models.

### In vitro models of diabetes

In vitro models of diabetes offer powerful tools for exploring the disease in a controlled laboratory setting. By isolating cells and tissues from the complexity of the whole organism, researchers can focus on the fundamental processes of insulin secretion, insulin resistance, and glucose metabolism with greater accuracy. These models are also widely used for drug discovery and testing, providing early insights into therapeutic potential before moving to animal studies or clinical trials. A concise comparative overview of the commonly used in vitro systems employed in diabetes research, along with their advantages and limitations, is provided in Table 3.

**Table 3:** Overview of widely used in vitro models for diabetes research. Each system offers unique strengths for studying aspects of β-cell biology, insulin resistance, and glucose metabolism, yet also carries limitations that must be considered when interpreting experimental outcomes

Model	Description	Advantages	Limitations	References
Pancreatic β-cell Lines (INS-1, MIN6, RIN-m5F)	Immortalized β-cell lines capable of glucose-stimulated insulin secretion.	Easy to culture; reproducible; suitable for high-throughput screening.	Phenotypic drift over passages; not fully identical to primary β-cells.	[107–109]
Isolated Islets of Langerhans	Primary pancreatic islets isolated from rodents or humans.	Retain native β-cell architecture; physiologically relevant insulin secretion.	Short lifespan ex vivo; variability between donors; technically demanding.	[110–112]
Adipocyte Cultures	Differentiated preadipocytes used to model insulin-regulated glucose uptake and lipolysis.	Useful for studying insulin resistance, lipid metabolism, and adipokine secretion.	Do not fully mimic in vivo adipose tissue complexity.	[113]
Hepatocyte Cultures	Primary or immortalized hepatocytes for studying hepatic glucose metabolism.	Model for hepatic insulin resistance, gluconeogenesis, and glycogen storage.	Rapid dedifferentiation in culture; limited lifespan of primary hepatocytes.	[114, 115]
Stem Cell-Derived β-like Cells	β-like cells generated from pluripotent stem cells that mimic pancreatic β-cell function.	Renewable source; human-specific model; potential for regenerative medicine.	Differentiation protocols are complex; functional maturity not identical to native β-cells.	[116–118]
Organ-on-Chip (OOC)	Microfluidic systems integrating pancreatic islets or β-like cells with perfusion and sensing components.	Recreates dynamic microenvironment; allows real-time functional monitoring; supports drug screening and multi-tissue interactions.	Material absorption, complexity, and lack of standardization; scalability remains limited.	[119–124]

#### Pancreatic β-cell lines

Immortalized pancreatic β-cell lines, such as INS-1, MIN6, and RIN-m5F, are widely used experimental systems for studying β-cell physiology and the mechanisms of diabetes. The INS-1 cell line, established from rat insulinoma, secretes insulin in response to glucose stimulation and serves as a robust model for testing β-cell signalling pathways, cytotoxicity of diabetogenic agents, and antidiabetic drug screening [107, 125]. Similarly, the MIN6 cell line, derived from transgenic mouse insulinoma, retains glucose-inducible insulin secretion and exhibits expression profiles resembling those of primary mouse islets [108, 126, 127]. RIN-m5F cells, derived from rat insulinoma, are widely used as models for studying insulin gene regulation and β-cell apoptosis (Table 4) [128]. Their stable growth and insulin producing phenotype make them a convenient in vitro tool for dissecting molecular pathways involved in β-cell survival and function. However, they exhibit relatively weak glucose responsiveness compared to more physiologically faithful lines, such as INS-1 and MIN6, which limits their use in studies on glucose-stimulated insulin secretion (GSIS) [129, 130].

**Table 4:** Comparative characteristics of commonly used pancreatic β-cell lines. The table summarizes origin, key features, applications, and limitations, highlighting both rodent-derived and human-derived pancreatic β-cell line models in diabetes research

Cell Line	Origin	Key Features	Applications	Limitations	References
INS-1	Rat insulinoma	Glucose-responsive insulin secretion; well characterized; good transfection efficiency	Insulin secretion studies, β-cell signalling, drug testing	Glucose responsiveness diminishes at higher passages; rodent origin	[125, 131]
MIN6	Mouse insulinoma	Robust insulin secretion; high glucose sensitivity; stable proliferation	β-cell gene regulation, apoptosis, insulin signalling	Require low passages; species differences with humans	[126, 127]
RIN-m5F	Rat insulinoma	Easy to culture; useful for gene regulation and apoptosis studies	β-cell apoptosis and gene expression research	Poor glucose responsiveness; low insulin content	[128]
HIT-T15	Hamster insulinoma	Secrete insulin in response to glucose and secretagogues	Early β-cell biology and insulin secretion studies	Rapid loss of glucose responsiveness upon passaging	[132, 133]
TC (e.g., TC-6, TC-3)	Mouse insulinoma	Produce insulin; moderate glucose responsiveness	β-cell signalling, tumour biology	Variable insulin secretion; less widely used today	[134–136]
EndoC-βH1/βH2	Human foetal pancreatic tissue, immortalized	Human β-cell–like phenotype; robust glucose-responsive insulin secretion; physiological relevance	Human-relevant diabetes research, drug screening, β-cell biology	Limited availability; costly; finite proliferation	[137–139]
βLox5	Human pancreatic tissue (immortalized)	Retain β-cell markers; moderate insulin secretion	Human β-cell studies, toxicology, drug testing	Heterogeneous population; limited glucose responsiveness	[139]

Recent studies have continued to leverage RIN-m5F cells for apoptosis and stress pathway studies. For instance, RIN-m5F cells were used to show that apolipoprotein C3 induced β-cell apoptosis via oxidative stress and that treatment with kaempferol mitigated this effect [109]. In another study, the role of connexin Cx30.2 in RIN-m5F cells under glucotoxic conditions was investigated, revealing that knockdown of Cx30.2 increased apoptosis under high glucose challenge [140]. Kornelius et al. (2018) demonstrated that liraglutide rescued RIN-m5F cells from glucolipotoxicity-induced apoptosis by restoring PDX1 expression [129]. Although RIN-m5F cells are less suitable for glucose-stimulated insulin secretion (GSIS) studies, they continue to be extensively utilized in mechanistic investigations of β-cell stress, signaling, and apoptosis. In addition to these cells, HIT-T15, TC, EndoC-βH1/βH2, and βLox5 have also been used in diabetes research (Table 4). All these β-cell lines are relatively easy to maintain, reproducible, and highly amenable to genetic manipulation, making them valuable tools in experimental research. However, their immortalized nature and phenotypic changes that occur with prolonged passaging reduce their ability to fully replicate the physiology of primary human β-cells.

#### Isolated islets of langerhans

Isolated islets serve as a versatile platform for exploring multiple aspects of diabetes biology. These models allow for a detailed exploration of β-cell function, such as glucose-stimulated insulin secretion, calcium signaling, and electrophysiological properties, while minimizing the confounding effects of systemic influences [110]. In addition, these models are often challenged with diabetogenic stimuli, such as pro-inflammatory cytokines, chronic hyperglycemia, lipotoxic conditions, or chemical toxins, replicating the molecular and cellular stressors that drive β-cell dysfunction in both T1DM and T2DM [111]. Beyond mechanistic studies, isolated islets are a valuable tool in drug discovery, offering a controlled system for screening pharmacological agents, incretin-based therapies, and natural compounds for their capacity to enhance insulin secretion and preserve β-cell survival [141]. Moreover, isolated islets play a pivotal role in transplantation and regenerative medicine research, as exemplified by the success of the Edmonton Protocol and recent advances in generating stem cell-derived islet-like organoids [112, 142]. Comparative studies across species, including rodents, pigs, and humans, have revealed distinct differences in islet physiology. These interspecies insights not only deepen our understanding of pancreatic biology but also help pinpoint mechanisms that are more likely to be clinically relevant [143].

Despite their unique advantages, the use of isolated islets has limitations. However, the isolation process can compromise islet integrity and viability, and their functional maintenance ex vivo is typically limited to short culture periods of approximately 7 to 10 days. Human islet studies are further constrained by donor-to-donor variability and limited tissue availability, factors that often hinder reproducibility and consistency across experiments [144]. Nevertheless, isolated islets remain an indispensable model, striking a crucial balance between reductionist cell line systems and the complexity of whole-animal studies. By providing a physiologically relevant yet experimentally accessible platform, these models continue to serve as a bridge between fundamental research in β-cell biology and translational efforts to better understand and treat diabetes. Looking forward, the integration of islet research with new technologies, such as stem cell-derived β-cells, organoid systems, and organ-on-chip (OOC) platforms, holds the potential to significantly boost their importance and accelerate the development of precision therapies [144, 145].

#### Stem cell-derived β-like cells

Stem cell-derived β-like cells have emerged as an innovative and promising model for studying diabetes and offer a renewable and physiologically relevant source of insulin-producing cells. Unlike immortalized β-cell lines, which often lack robust glucose responsiveness, stem cell–derived models provide an opportunity to generate functional β-like cells that closely mimic the phenotype of native human pancreatic β-cells. These systems hold enormous potential for dissecting the mechanisms of β-cell development, function, and failure, while also providing platforms for regenerative therapies and drug discovery [116, 117].

The generation of β-like cells typically relies on the directed differentiation of human embryonic stem cells (hESCs) or induced pluripotent stem cells (iPSCs) through stepwise protocols that recapitulate embryonic pancreatic development [116]. These protocols involve staged exposure to growth factors and small molecules, guiding pluripotent cells through the definitive endoderm, pancreatic progenitor, and endocrine precursor stages before terminal differentiation into insulin-expressing cells [146]. While early protocols produced immature β-like cells with limited glucose responsiveness, refinements in culture conditions, three-dimensional organoid systems, and maturation strategies have significantly improved their functional properties, including glucose-stimulated insulin secretion and electrophysiological responsiveness [118].

Stem cell–derived β-like cells have diverse applications as experimental models. They enable a detailed analysis of the molecular pathways governing β-cell specification and maturation, providing insights into diabetes-associated genetic variants and developmental defects. They are increasingly used to study the mechanisms of β-cell dysfunction and death under diabetogenic stressors, such as inflammatory cytokines, lipotoxicity, and glucotoxicity, offering a human-relevant system that complements rodent islet models [118, 146]. Importantly, patient-derived iPSCs allow the generation of β-like cells carrying disease-specific genetic backgrounds, enabling personalized modelling of monogenic and polygenic forms of diabetes [147, 148]. Furthermore, stem cell–derived β-like cells are essential platforms for drug discovery, enabling the identification of compounds that enhance β-cell survival, proliferation, and insulin secretion under diabetogenic stress. Despite these advances, significant challenges remain, particularly in achieving full functional maturity comparable to that of native human β-cells, ensuring durable graft survival after transplantation, and overcoming immune rejection in clinical applications.

### Islets-on-chip

Organ-on-chip (OOC) technologies are becoming increasingly influential tools in diabetes research, striking a distinctive balance between simplified in vitro models and intricate in vivo systems (Table 5). By combining microfluidics with living cells, these devices simulate the dynamic features of the islet microenvironment, such as nutrient and oxygen gradients, shear stress, and vascular-like perfusion, which are absent in static cultures. Such systems enable real-time monitoring of β-cell function, insulin secretion kinetics, calcium flux, and metabolic activity under physiologically relevant conditions [119, 149]. Importantly, OOC devices also sustain islet viability for extended periods, facilitate the modeling of diabetogenic stressors such as hyperglycemia or lipotoxicity, and permit coupling with other tissues such as liver or vasculature to study systemic interactions [120, 123, 150]. Islet-on-chip systems are being increasingly utilized not only for disease modeling but also for drug testing and assessing stem cell-derived islet-like cells before transplantation, thus accelerating translational applications [119]. Although challenges remain, including material limitations, standardization, and scalability, continued innovation in chip design, biosensors, and integration with stem cell and organoid technologies positions islets-on-chip platforms as valuable tools for advancing diabetes research and precision medicine.

**Table 5:** Some of the studies of OOC platforms applied in diabetes research. The table highlights different chip designs, their main applications, and key findings, with emphasis on their use in modeling islet physiology, drug testing, and disease-relevant mechanisms

Chip / System Type	Main Goal / Application	Key Findings / Use in Drug Testing / Disease Modelling
Thermoplastic micro-physiological pancreas-chip	To model pancreatic islet structure/function with real-time readouts	Demonstrated automated glucose cycling, oxygen sensors, and assessed effects of antidiabetic medications on insulin secretion kinetics [121].
Islets-on-Chip (CHIP system)	Quality ranking and dynamic testing of human islet preparations	Developed a CHIP-score system where islet responses to glucose, GLP-1, and sulfonylureas (e.g. glibenclamide) were tested on-chip with electrophysiological and secretion readouts [119].
Microfluidic islet-on-chip / micropillar system	To recreate 3D islet microenvironment and test function	A micropillar based microfluidic platform supported 3D islet culture and allowed stimulation and analysis of β-cell function under flow [122].
Two-organ (islet and liver) microfluidic coupling	Recapitulate insulin–glucose feedback between pancreas and liver	Human islets in one chamber secreted insulin in response to glucose, stimulating glucose uptake in hepatic spheroids in the coupled chamber; this mimicked physiologic crosstalk [123].
Gradient-generator islet chip (engineered microenvironments)	To apply controlled concentration gradients and perfusable 3D arrays	Created a chip with gradient generator and 3D culture array to test how variable concentrations of glucose or other compounds influence islet behaviour [124].
Multi-organoid-on-chip (hiPSC derived)	Model liver-islet axis in a human-derived system	Used hiPSC derived islet and liver organoids in a linked platform to simulate metabolic interactions and test perturbations [151].
hPSC-derived islet organs via microfluidic device	Combine differentiation and screening in-chip	Built a multi-layer microfluidic device enabling in situ aggregation and differentiation of iPSC-derived islet organoids; potential for drug screening [152].

## Conclusions

Experimental models of diabetes remain indispensable tools for unravelling the complex mechanisms underlying the onset, progression, and complications of the disease. From in vivo systems such as diet-induced, chemically induced, genetic, and surgical models to in vitro approaches including immortalized β-cell lines, isolated islets, adipocyte cultures, and stem cell–derived β-like cells, each model provides unique insights into the different facets of diabetes pathophysiology. Although no single model can fully recapitulate the multifactorial nature of human diabetes, their complementary strengths enable researchers to address specific mechanistic questions, evaluate therapeutic strategies, and bridge the gap between fundamental science and clinical application. Emerging technologies, particularly stem cell–derived and patient-specific models, hold promise for personalized medicine, offering platforms that closely mirror human physiology and genetic diversity. Moving forward, the thoughtful integration of traditional and advanced models will be essential to accelerate the discovery of effective interventions, improve translational relevance, and ultimately reduce the global burden of diabetes.

Future advances in diabetes research will depend on models that faithfully reflect the complexity of human diseases. Innovative platforms such as 3D organoids, organ-on-chip systems, and patient-specific cells derived from iPSCs, provide more physiologically relevant environments and open the door to personalized insights into disease mechanisms and treatment responses. When combined with powerful tools such as gene editing and artificial intelligence-driven analysis, these approaches have the potential to accelerate drug discovery, improve the translation of research into clinical practice, and move us closer to true precision medicine in diabetes care.

## Data Availability

Not applicable.

## Abbreviations

ARC: Arcuate Nucleus
ATP: Adenosine Triphosphate
DPP-4: Dipeptidyl Peptidase-4
ER: Endoplasmic Reticulum
fa/fa: Zucker Fatty Rat (homozygous mutation)
GK: Goto-Kakizaki (Rat)
GLP-1: Glucagon-Like Peptide-1
GSIS: Glucose-Stimulated Insulin Secretion
hESCs: Human Embryonic Stem Cells
HFD: High-Fat Diet
HFFD: High-Fat, High-Fructose Diet
HFHS: High-Fat, High-Sucrose Diet
HLA: Human Leukocyte Antigen
Idd: Insulin-Dependent Diabetes (Susceptibility Loci)
IDF: International Diabetes Federation
INS-1: Rat Insulinoma Cell Line
iPSCs: Induced Pluripotent Stem Cells
Lep: Leptin Gene
MIN6: Mouse Insulinoma Cell Line
MLD-STZ: Multiple Low-Dose Streptozotocin
MSG: Monosodium Glutamate
NAD⁺: Nicotinamide Adenine Dinucleotide
NAFLD: Non-Alcoholic Fatty Liver Disease
NOD: Non-Obese Diabetic (Mouse)
PARP: Poly (ADP-ribose) Polymerase
RIN-m5F: Rat Insulinoma Cell Line
ROS: Reactive Oxygen Species
STZ: Streptozotocin
T1DM: Type 1 Diabetes Mellitus
T2DM: Type 2 Diabetes Mellitus
VMH: Ventromedial Hypothalamus
